# Applying Gel-Supported Liquid Extraction to Tutankhamun’s Textiles for the Identification of Ancient Colorants: A Case Study

**DOI:** 10.3390/gels9070514

**Published:** 2023-06-25

**Authors:** Greta Peruzzi, Alessandro Ciccola, Adele Bosi, Ilaria Serafini, Martina Negozio, Nagmeldeen Morshed Hamza, Claudia Moricca, Laura Sadori, Gabriele Favero, Valentina Nigro, Paolo Postorino, Roberta Curini

**Affiliations:** 1 Institute for Complex System, National Research Council, Sapienza University, Piazzale Aldo Moro 5, 00185 Rome, Italy; 2 Department of Physics, Sapienza University of Rome, Piazzale Aldo Moro 5, 00185 Rome, Italy; paolo.postorino@roma1.infn.it; 3 Department of Chemistry, Sapienza University of Rome, Piazzale Aldo Moro 5, 00185 Rome, Italy; adele.bosi@uniroma1.it (A.B.); ilaria.serafini@uniroma1.it (I.S.); roberta.curini@uniroma1.it (R.C.); 4 Department of Earth Sciences, Sapienza University of Rome, Piazzale Aldo Moro 5, 00185 Rome, Italy; nagmeldeenmorshed.hamza@uniroma1.it; 5 Department of Environmental Biology, Sapienza University of Rome, Piazzale Aldo Moro 5, 00185 Rome, Italy; negozio.1920109@studenti.uniroma1.it (M.N.); claudia.moricca@uniroma1.it (C.M.); laura.sadori@uniroma1.it (L.S.); gabriele.favero@uniroma1.it (G.F.); 6 Grand Egyptian Museum, Conservation Center, Al Remaya Square, Giza 3513204, Egypt; 7 ENEA C.R. Frascati, Fusion and Technologies for Nuclear Safety and Security Department, Via E. Fermi 45, 00044 Frascati, Italy; valentina.nigro@enea.it

**Keywords:** SERS, dyes, gel, micro-extraction, non-invasive, archaeological textile

## Abstract

The identification of the dyes present on a linen fragment from the tomb of Pharaoh Tutankhamun is the objective of the present study. Fiber optic reflectance spectroscopy (FORS) was applied to the archaeological sample for preliminary identification of the dyes and to better choose the extraction methodology for different areas of the sample. The innovative gel-supported micro-extraction with agar gel and the Nanorestore Gel^®^ High Water Retention (HWR) gel were applied to the archaeological sample after testing of the best concentration for the extraction of the agar gels substrates, performed on laboratory mock-ups by means of UV–Vis transmittance spectroscopy. Immediately after extraction, Ag colloidal pastes were applied on the gel surface and Surface Enhanced Raman Scattering (SERS) analysis was performed directly on them. The combination of information deriving from FORS and SERS spectra resulted in the successful identification of both indigo and madder and, in hypothesis, of their degradation products.

## 1. Introduction

The use of dyed threads or dyed clothes in ancient Egypt could tentatively be traced back to the First Dynasty (3150–2925 BC), but it is only from the New Kingdom onwards (18th Dynasty, 1543–1292 BC) that cloth woven with colored thread was increasingly employed [1]. The most used fiber was linen and since not everyone could afford high quality linen, thread was often dyed using ochres rather than plant dyes [2]. The first was indeed utilized for dyeing in brown or dark red shades by hydrating iron oxide and mixing it with clay. Vibrant red textiles were obtained through the use of Mediterranean plants such as madder (*Rubia tinctorum*) or alkanet (*Alkanna tinctoria*) [3,4]. Yellow tones, instead, were achieved from safflower (*Carthamus tinctorius*) or turmeric (*Curcuma longa*), while indigo (*Indigofera tinctoria*) was the main source of blue colors. Other shades, such as green colors, were obtained by mixing blue and yellow dyestuffs [2,4].

In recent decades, the interest in studying organic dyestuff historically used to dye textiles has grown. These objects, in fact, possess great cultural importance and, in particular, the study of organic colorants allows for achieving historical and technological information about manufacturing, trades, exchanges, and civilization evolutions. However, the analysis of historical textile samples represents a complex challenge from the analytical point of view due to the usually limited amount of samples available, the low concentration of chromophores in the original material, and the presence of possible degradation products [5]. Textile fibers are generally susceptible to degradation mechanisms involving physicochemical and, consequently, mechanical processes that are eventually caused by microbiological attacks. These can result in the integrity loss of the fiber itself. The dye, in turn, can be subjected to photo-oxidative reactions, leading to fading and alteration of the original chromatic features [6]. Consequently, the place and methods of preservation of textile are fundamental for defining its current state of conservation. From this point of view, the archaeological contexts could represent “extreme” situations in which the microbiological attack is disadvantaged. In several cases (e.g., deserts, acidic peat bogs, alkaline lake muds, and perennial ice [7]), it is not uncommon to find remains of dyed fabric preserved up to the present day. Textile artifacts that survive are therefore extremely valuable; the definition of the dyeing matrices and the technologies employed to make the object not only help in reconstructing ancient cultures but also play a crucial role in developing and fine-tuning specific conservation methods.

Consequently, in the field of chemistry applied to cultural heritage, the analysis of the dye composition represents a powerful instrument, but it is also one of the most interesting analytical challenges. High pressure liquid chromatography coupled with an appropriate detector (diode array, HPLC-DAD; mass spectrometer, HPLC-MS) remains the most reliable and versatile identification method for organic colorants today [5,8,9,10,11], while the identification of dyes directly on fabrics without separative methods is often complex because of the organic matrix, which interferes in most analytical techniques. Nonetheless, when dealing with objects of art, the application of techniques which require sampling is discouraged and a multi-technical approach which includes both non-invasive and micro-invasive techniques is always preferred. During the last decades, great effort has been undertaken to develop minimally invasive techniques with increased sensitivity. In this sense, fiber optic reflectance spectroscopy (FORS) and hyperspectral imaging in the UV–Vis–NIR range have been demonstrated to be efficient tools for the rapid, non-invasive, in situ preliminary characterization of many artistic materials [9,12,13,14,15,16,17,18]. Additionally, Raman and Surface Enhanced Raman Scattering (SERS) spectroscopies have attracted the interest of many research groups for their ultrasensitive and high detection capability. In particular, in the latter case, SERS spectroscopy has proved to be a valid technique for the characterization and study of organic dyes [19,20,21,22,23,24]. Indeed, by exploiting metallic substrates, such as, for example, silver nanoparticles, it is possible to amplify the Raman signal significantly, and this enhancement allows for overcoming the problem of strong fluorescence emission—typical of organic compounds—due to a localized surface plasmon resonance (LSPR) phenomenon, which comes into play when the incident light has the same vibrational frequencies of the valence electrons in the metal nanoparticle [25,26]. In this perspective, the use of gel substrates for the micro-invasive extraction of dyes and their consequent analysis by the SERS technique has recently been applied to the study of cultural heritage [27,28,29,30,31]. The most used in this sense is the agar gel because it favors the interaction of silver nanoparticles with consequent enhancement of the SERS signals due to the shrinkage of its structure after drying [28]. For example, in 2015, Platania and colleagues [32] presented a methodology for the extraction and detection of indigo dyes in painting and textiles involving Ag-agar gel soaked into a reducing solution, resulting in a safe procedure for both laboratory samples and works of art. Despite this, other types of gels have been used, such as, for example, the Nanorestore Gel^®^ High Water Retention (HWR) gel—patented for cleaning surfaces—which was tested for the first time by Germinario in 2020 for the extraction of dyes from textiles [33]. Both agar and Nanorestore Gel^®^ were employed in the aforementioned work for the extraction of madder and cochineal from wool mockups using the state-of-the-art ammonia-based solution devised in 2016 by Lombardi and colleagues [34]. This methodology, stated as ‘mild’, tested, for the first time, a basic environment using ammonia, Na_2_EDTA, and NaCl for the extraction of anthraquinones in order to preserve glycosylated moieties, which are sensitive and may be lost in traditional acidic methodologies. The work by Germinario, through a multi-technical approach, proved highly successful for SERS analysis, allowing discrimination between madder and cochineal on both gels. The research was pushed forward and the methodology was revised and implemented for hydrophilic paint layers in the study by Bosi [30], where agar at different concentrations (ranging from 1% to 12%) and Nanorestore Gel^®^ were tested for the extraction of madder lake pigments showing positive outcomes in terms of non-invasiveness. Agar gel concentrations below 4% permitted the extraction without ripping the paint and did not show any color change on the mockups. From the operative point of view, moreover, the methodology (which is defined as “gel-supported liquid extraction”) is even very simple. Briefly, the gel is cut into cylinders and soaked in the extraction solution for a certain amount of time. Then, these substrates are removed with tweezers and put in contact with the sample surface in order to extract the analytes present on the surface [30]. This makes this approach suitable for different typologies of laboratories.

For all these reasons, in the present work, we decided to investigate more deeply the behavior of extraction of agar gel in the range of concentrations between 2% and 4%. In this way, in comparison to the previous literature, a deeper insight was accomplished in order to individuate the agar gel concentration, coupling the best extraction performances to handing features for the analytical methodology [30]. Agar gel was tested along with Nanorestore Gel^®^ on both paint and textile laboratory mock-ups tinted with madder and indigo; spectroscopic analysis including UV–Vis and Raman SERS spectroscopy were performed on these samples and also served as a reference spectral database. In this way, the methodology was hence applied to a wider set of artist matrices (for instance, the tempera mock-ups) and with a systematic approach in comparison to our previous works [30,33]. It was also employed for the diagnostics of a precious sample, an archaeological textile fragment from Tutankhamun’s tomb. Preliminary spectroscopic analysis was conducted on the archaeological sample using FORS to understand which extraction procedure should be followed. Gels were then soaked in two different solutions according to the area of extraction (blue or red), Ag-colloidal pastes were applied immediately after extraction, and SERS analysis was performed to characterize the dyes. The combination of gel-supported liquid extraction with the use of colloidal paste represents a different approach in comparison to the previous studies [30,33,35], which takes advantage from the formation of extended nanoclusters for the enhancement of the Raman signal. The application of this sample allowed for achieving an actual valorization in a real case study, and it integrated the gel-supported liquid extraction in an analytical protocol that also involved non-invasive techniques: this promotes the transferability of the new technology in routine cultural heritage diagnostics.

## 2. Results and Discussion

### 2.1. UV–Vis Spectroscopy

**Figure 1:**
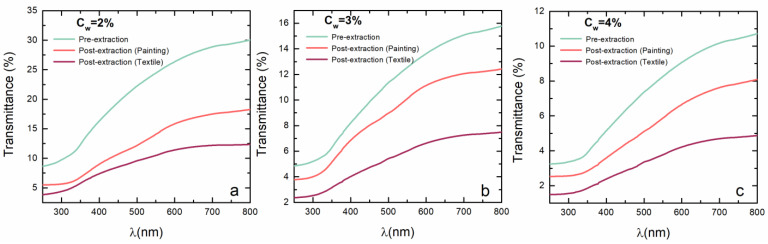
UV–Vis transmittance spectra of agar gel at (**a**) C_w_ = 2%, (**b**) C_w_ = 3%, and (**c**) C_w_ = 4% of the gel before extraction (green), after extraction from the madder paint mockup (orange), and after extraction from the madder textile mockup (red).

The UV–Vis spectra of agar gel in the concentration range between 2% and 4% have been compared to determine the concentration of agar that is the most appropriate to employ for the extraction of the real case study. The spectrometric measurements of agar are taken before the extraction and after the extraction both on the paint and textile mock-ups. In Figure 1, the transmittance spectra at three different concentrations (2%, 3%, 4% in water *w*/*w*) are reported. For all tested concentrations, the transmittance is higher in the samples pre-extraction than in the samples post-extraction. Furthermore, the spectra of agar gel after the extraction on the textile sample show lower transmittance values, probably due to the higher absorption of incident radiation of the extracted dye. The UV–Vis spectra of agar gel exhibit the same pattern at all concentrations after being soaked in the ammonia solution, along with Na_2_EDTA and NaCl. Up until 300 nm, transmittance values are constant, then they start to quickly rise. The spectra of agar gel after extraction on the paint mock-up show a wide absorption band between 480 nm and 550 nm, which is due to the electron transitions *n* → π* of carbonyl groups present in the chromophore alizarin [36]. Samples with 2% and 3% of agar gel post-extraction are more susceptible to the UV–Vis radiation than samples at C_w_ = 4%, with a more evident decrease in transmittance around 400 and 500 nm, which is due to the typical absorbance bands of madder, as shown in Figure 1. Spectra of agar gel after the extraction on the textile mock-up show slight absorption characteristic bands that confirm the major capability of extracting the dye from paint rather than textile. Nevertheless, when comparing all the concentrations, 2% agar gel and 3% agar gel clearly show the absorption band opposed to all other tested concentrations.

An evaluation of the invasiveness of the procedure for the mock-ups was performed by means of optical analysis and FORS-colorimetry. The observation at the microscope did not evidence gel residues on the surface of the mock-ups and no observation about the morphology of the mock-up was observed. From the point of view of color changes, the calculation of color variation ΔE_00_ using the CIEDE2000 formula resulted in values lower than three, which were considered as the upper limit of rigorous color tolerance. With the reference to these results, the methodology can be considered remarkably micro-invasive because it extracts the analytes without causing damage or visible color variations on the artist matrices. However, with reference to more sensitive materials, further details about these aspects can be found in previous works [30].

### 2.2. Case Study: Archaeological Textile Fragment from Tutankhamun’s Tomb

#### 2.2.1. Gel Micro-Extraction In Situ

Based on tests conducted on laboratory mock-ups and results obtained with UV–Vis spectroscopy, it was decided to use 3% agar gel for the extraction in the real case study as it showed the best results in terms of extraction capacity without leaving any residue.

**Figure 2:**
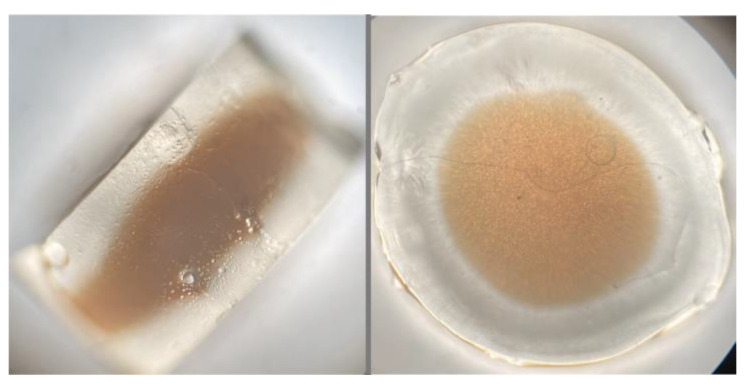
Optical microscope (4× magnification) images of Nanorestore Gel^®^ soaked with the reducing extraction solution used for indigo extraction.

Both agar gel and Nanorestore Gel^®^ worked well with the ammonia-based solution for the extraction of the red dyes. On the contrary, in the blue area, it was possible to use only agar gel since Nanorestore Gel^®^ appeared to be incompatible with the indigo reducing solution. Further evaluations must be performed in this aspect since it could be interesting to understand if the solution causes polymer degradation rather than sodium dithionite concrete forming inside the gel (Figure 2).

#### 2.2.2. Fiber Optic Reflectance Spectroscopy (FORS)

**Figure 3:**
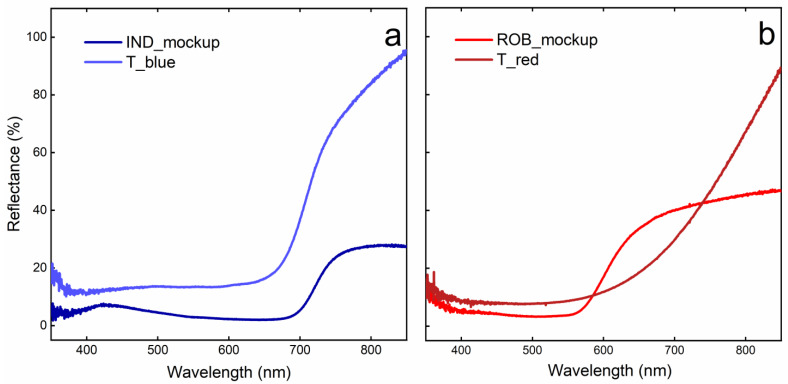
(**a**) Comparison between FORS spectra of indigo mockup (dark blue) and of the blue area of the archeological sample; (**b**) comparison between FORS spectra of madder mockup (red) and of the red area of the archaeological fragment (Bordeaux).

FORS analyses on the archaeological samples were useful to hypothesize a first characterization of the dyes present. Indeed, by comparing spectra acquired on the bluish area and that of the indigo mockup (Figure 3a), it is possible to underline spectral similarities in the 650–700 nm region where a broad absorption band typically attributed to the π → π* of the C=C double bond is present [37]. Moreover, through applying the first derivative, it is possible to observe a flex around 700 nm, which is perfectly in line with what is reported in the literature for indigo and woad [37]. However, it is important to highlight that the weak maximum in the violet region, observable in the mock-up and cited in the literature, is not visible in the spectrum of the archaeological sample [37].

Quite the opposite is evident when comparing the results obtained from the reddish area with the reference spectra of red dyes and laboratory mockups (Figure 3b, showing the comparison with a reference mock-up of madder mockup). Indeed, the presence of a wide absorption band between 400 and 700 nm disallows any attribution to typical spectral features of known colorants of reddish shade.

#### 2.2.3. Surface Enhanced Raman Scattering (SERS)

**Figure 4:**
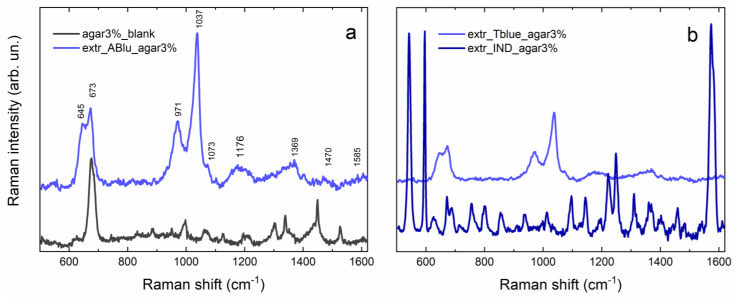
(**a**) Comparison between SERS spectra of 3% agar blank (gray) and 3% agar after extraction of the blue area (light blue); (**b**) comparison between SERS spectra of 3% agar after extraction of the indigo mockup (dark blue) and 3% agar after extraction of the blue area (light blue).

Results obtained from SERS spectra acquired on agar gel after extraction (Figure 4a) confirm the presence of an indigo dye on the blue area of the Tutankhamun’s fragment. Nonetheless, by comparing results obtained from the extraction of the archaeological sample and those from the indigo mockup (Figure 4b), the absence of the most intense signals of indigo is evident for the former, although characteristic peaks of both indigotin (1073 w, 1176 vw, 1369 w, 1470 vw) and indirubin (645 m, 971 m, 1404 w, 1585 vw) are present. Indeed, signals of indirubin are generally more intense than those of indigotin, probably due to thermal degradation of the dye [38]. In particular, peaks at 645, 971 and 1404 cm^−1^ can be attributed to bending modes of the C-C, C-H, and N-H bonds of indirubin [39], while the signal at 1073 cm^−1^ is attributed to a ring stretching and C-O rocking of indigotin [32]. The broad band centered at 1176 cm^−1^ also refers to indigotin and corresponds to C-C stretching and C-H bending [32]. The band at 1585 cm^−1^, instead, can be attributed to stretching modes of the C-C, C=O, and C=C bonds of indirubin [39]. Finally, the peak at 673 cm^−1^ is probably due to agar gel, as proven by the comparison in Figure 4a, while the very intense signal at 1037 cm^−1^ has been hypothesized to be a degradation product, probably anthranilic acid, which is a known degradation product of indigotin. Indeed, according to Poulin [40], one of the main degradation products of indigo is isatin, which is formed when indigotin oxidizes. If the degradation goes further and a secondary reaction takes place, anthranilic acid is formed. In 2014, Chadha and colleagues reported a very intense SERS band at 1036 cm^−1^ attributed to the phenolic ring bending and to the NH_2_ rocking of anthranilic acid [41]. The results about the strong affinity between anthranilic acid and the Ag-NPs reported in the aforementioned work support both the hypothesis of the presence of anthranilic acid as a degradation compound on the blue area of the Tutankhamun’s textile fragment and also justify the relatively strong intensity of the SERS signal we observed. However, further analysis through HPLC/MS could help to clarify the nature of this compound.

**Figure 5:**
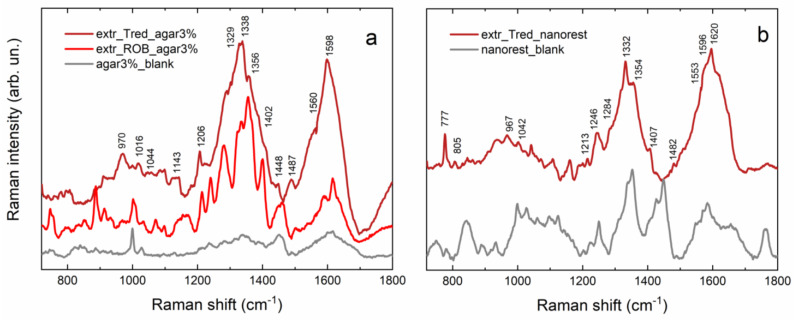
(**a**) Comparison between SERS spectra of 3% agar gel blank (gray), 3% agar gel after extraction from madder mockup (light red), and 3% agar gel after extraction from red area of archaeological sample (Bordeaux); (**b**) comparison between SERS spectra of Nanorestore Gel^®^ blank (gray) and Nanorestore Gel^®^ after extraction from red area of archaeological sample (Bordeaux).

SERS analysis performed on gels after extraction of the red area of Tutankhamun’s fragment gave good quality spectra for both agar (Figure 5a) and Nanorestore Gel^®^ (Figure 5b). In both cases, it is possible to recognize the characteristic peaks of madder; most notably, by comparing the spectrum acquired on agar gel after extraction from the archaeological sample and from the madder mockup, a clear correspondence between the peaks is observed (Figure 5a). In particular, the peak at 1448 cm^−1^ is present in the spectrum acquired on agar and is attributable to C-O stretching, C-O-H bending, and C-H bending [20,28,42]; whereas, the peak around 1400 cm^−1^, which is a characteristic signal of purpurin, is very clearly seen in the spectrum acquired on the Nanorestore Gel^®^. Furthermore, the band around 1330 cm^−1^ present in both spectra is typical of madder and refers to the content of alizarin and purpurin, while the shoulder at 1290 cm^−1^ and the band around 1590 cm^−1^ are typical signals of anthraquinone molecules and correspond to the C-C and C=O stretching of the ring, respectively [28]. Results for madder were eventually confirmed and supported by LC/MS data obtained after re-extraction from the gels, whose results are the subject of another publication by the same authors.

## 3. Conclusions

The multi-technical approach pursued in this research study, composed of gel microextraction and the subsequent spectroscopic characterization, enabled the detection of the dyes present on Tutankhamun’s textile fragment without posing any threat to the artifact.

First, tests conducted on laboratory mock-ups permitted to investigate how different concentrations of agar gel and UV–Vis transmittance spectroscopy have contributed to the choosing of 3% agar gel as the suitable concentration.

The application of the FORS technique on the archaeological sample was useful to obtain preliminary data in a totally non-invasive way and to consequently hypothesize the composition of the dyes present. This was true for the bluish area where the presence of an indigoid compound was even supported by the comparison with a reference spectrum of indigo. On the contrary, for the reddish area, the reflectance spectrum did not allow us to retrieve any information because of the wide absorption in the visible light range.

The application of the gel-supported liquid extraction protocol allowed a micro-sampling of the dyes both on the blue and red areas. The consequent detection was performed by SERS spectroscopy directly on the gel by contact of its surface with a silver colloidal paste. The SERS spectra confirmed the presence of indigo dye, from the characteristic signals of indirubin and (with lower intensity) indigotin, while, for the red area, it was possible to observe the characteristic signals of madder dye by comparison with reference spectra of the madder mockup. It is interesting to evaluate the potential and the complementarity of SERS applied to the gel extraction in comparison to FORS. For the indigo dye, the combination of FORS and SERS was useful in effectively detecting the presence of the blue colorants and in providing information about degradation processes. While FORS suggested the presence of indigotin, the SERS data results were indicative of decomposition products. In the case of madder, FORS could not provide information for the dye identification, and only the on-gel approach allowed a clear identification of the chromophores. These aspects highlight the effectiveness and the information potential resulting from the gel-supported liquid extraction methodology.

It is fundamental to mention some aspects of the study, which require further research. At first, in this work, we limited the testing only to one typology of laboratory-made gel and to one parameter, the polymer/gel concentrations, but deeper studies would be necessary for better optimization of the gel-supported liquid extraction methodologies. Further gels could be studied, and the influence of other aspects (for instance, the thickness of the gel) should also be evaluated. The final protocol adopted for the historical sample requires integrating further analytical techniques, such as HPLC/MS, in order to provide the complete characterization of the dye chromophores and their final characterization. These aspects are the object of a future publication. Finally, regarding the hypotheses about degradation products observed by means of SERS, further analyses must be performed in order to confirm the presence of anthranilic acid.

## 4. Materials and Methods

### 4.1. Commercial Products

For the preparation of mock-ups, madder roots (*Rubia tinctorum* L.) and alum were purchased from Chroma Srl (Milano, Italy), while indigo in powder (*Indigofera tinctoria* L.) was purchased from Kremer Pigmente (Berlin, Germany). Cream of tartar (99.9%) and sodium carbonate (99.9%) were purchased at a local grocery shop.

Agar in powder (ash 2.0–2.4%), solvents, and salts such as K_2_CO_3_ (with impurities ≤55.0 ppm), ammonia (30–33%), NaOH (≥95%), hydroxylamine hydrochloride (99.9%), NaCl (with impurities ≤0.005% as insoluble matter), Na_2_EDTA (with impurities ≤0.005% as insoluble matter), and silver nitrate (≥99.0%) were purchased from Sigma-Aldrich (Burlington, MA, USA). The Nanorestore Gel^®^ High Water Retention Gel is produced by the Italian Center for Colloids and Surface Science (CSGI); information about this kind of gel is available on the Nanorestore Gel^®^ High Water Retention Gel technical data sheet and in the previous literature [43,44,45,46,47,48].

### 4.2. Mock-Ups Preparation

A paint mock-up was prepared following traditional procedures reported in the literature [49]. First, preparation was performed by soaking 17 g of animal glue in 250 mL of water and leaving it overnight. The glue was then heated up at 45 °C until completely melted. Later, approximately 100–150 g of gypsum was added to the solution, which was applied on a brick in eight perpendicular coats. The whole was left drying for one week and then polished with sandpaper to make the surface smooth and homogeneous. The madder lake pigment was prepared following a recipe from Daniels et al. [49]. In brief, 5 g of madder roots were soaked in 150 mL distilled water and left overnight. The roots in water were heated up to 70 °C for 30 min. After filtration, 2.5 g potassium alum was added to the solution and the temperature was brought to 80 °C. Meanwhile, 0.94 g K_2_CO_3_ was dissolved in 25 mL water and gently poured into the dye bath under continuous stirring. The lake pigment was left precipitating overnight, filtered, and ground. Lake pigment was hence mixed with egg yolk and applied in layers on the previously prepared brick.

Laboratory textile mock-ups were prepared by wrapping and compacting dyed wool yarn around a microscope slide to simulate the surface of a fabric. For the dyeing process, ancient recipes already described in the literature and historically employed for dyeing with natural dyestuffs were followed [4,33,34]. Concerning madder, the procedure was divided into two steps: mordanting and dyeing. The mordanting bath was prepared by mixing 310 mg of alum and 60 mg of cream of tartar in 250 mL of distilled water and the solution was heated up to 40 °C for ten minutes. Then, it was left to cool to 25 °C before adding 1 g of raw purged wool into the bath. Following this, the temperature was slightly raised to 80 °C over a period of 40 min, and the wool was maintained in the bath at this temperature for 1 h under gentle magnetic stirring. After that time, the bath was cooled at room temperature for over 20 min and the yarn was squeezed out and left to dry. A dyeing bath was prepared by soaking 1 g of crumbled madder roots in 400 mL of distilled water. The mordanted wool was added to a lukewarm bath while the temperature was increased to 80 °C over a period of 40 min and kept for 1 h under gentle magnetic stirring. Subsequently, the wool was left cooling in the bath for 30 min, then squeezed and washed repeatedly until the water was completely clear. Finally, it was left to dry.

The mechanism for dyeing with indigo involves a redox reaction (vat dyeing) in which indigo—which is usually insoluble in water—is reduced to its soluble leuco-form (leuco indigo) in alkaline conditions [4,50]. This allows the dye to penetrate into the fibers; the final color is reached and maintained through oxidation during the drying process, which makes indigo insoluble in water and impossible to be washed out [50]. A dyeing bath was hence prepared by mixing 0.6 g of minced indigo powder in 10 mL of distilled water previously warmed at 45 °C. Then, a solution of 0.6 g of sodium carbonate dissolved in 6 mL of water and a solution of 1.5 g of sodium dithionite dissolved in 50 mL of lukewarm water (40–50 °C) were added. The whole solution was thus heated up to 55 °C and left at this temperature for 20 min. After that time, 3 g of raw purged wool was soaked into the bath and left for 10 min. The yarn was then extracted from the bath, squeezed, and left to air dry in order to allow the indigo to oxidize again and reach the final color. Finally, the wool was rinsed with distilled water until it was clear and then left to dry.

### 4.3. Archaeological Sample: Textile Fragment from Tutankhamun’s Tomb

After collecting and cataloging the most valuable remains from the tomb of Pharaoh Tutankhamun, the archaeologist Howard Carter swept the remaining materials from the surfaces of the tomb and deposited them in a wooden box. The box was closed in 1933 and stored in the Egyptian Museum in Cairo until 2017. One year later, it was moved to the Grand Egyptian Museum in Giza (Egypt), where its materials started to be subjected to scientific analyses.

Fragments presented here are linen-dyed textile pieces dating back to 1325 BC, the year of Pharaoh Tutankhamun’s death. They are part of a wider collection of textile objects (more than 750) discovered in the tomb and constitute the sole surviving royal wardrobe from the pharaonic period. This large number of textiles offers a significant glimpse into the use of fabrics in ancient Egypt, particularly during the 18th Dynasty. The textile under examination is woven with the tapestry technique, a distinctive method of weaving that incorporates decorative designs using colored threads on a loom. In particular, the fragments presented here have some tinted blue and red areas with some striped sections with both colors in them. More information about the sample can be found in the Figures S1–S3 and in previous publications [51].

### 4.4. Gel Preparation and Micro-Extraction In Situ

The micro-extraction in situ was carried out with two different kinds of hydrogels: agar gel and Nanorestore Gel^®^ High Water Retention (HRW) [33]. In previous studies, different concentrations between 1% and 12% have been tested with the best results for dye extraction observed at concentrations below 4% [9]. Therefore, here, we tested different concentration between 2% and 4% (2%, 2.5%, 3%, 3.5%, 4% in water *w*/*w*) of agar gel on naturally dyed wool and paint layer mockups before leading extraction on real case study samples. In brief, 0.16, 0.20, 0.24, 0.28 and 0.32 g of agar powder were dissolved in 8 mL of water, respectively, in three different beakers properly chosen to obtain a suitable thickness (~2 mm). The solution of agar was then heated up in a bain marie at 100 °C for 10 min and then cooled down for half an hour; afterwards, gels were stored in the fridge overnight before use. The Nanorestore Gel^®^ was utilized as a factory product. For UV–Vis analysis, agar gels at different concentrations were cut in squares with 2 cm sides, while, for SERS measurements, the extraction was performed using both Nanorestore Gel^®^ and agar gel cut in small cylinders (cylinders were cut in half for the extraction of the archaeological sample because of the fragments’ dimensions for a final size of about 3.5–4 mm of diameter) obtained with the back of a Pasteur pipette and loaded in the respective extracting solutions for 90 min [33].

For the extraction of the red area, we prepared a solution of 1 mM NH_3_/Na_2_EDTA (1:1) with 4.7 mM NaCl following the procedure pointed out in [34]. Both agar and Nanorestore Gel^®^ were thus applied on the surface of the red area after the gel had lost 5% of its weight and it was left in extraction for 3 h. On the blue area, instead, the dye was extracted using a reducing solution containing NaOH/Na_2_S_2_O_4_ (1:2) dissolved in water [32]. Gels were subsequently applied directly on the area of extraction at 100% of their weight but the exceeding solution was removed using adsorbent paper and letting it absorb the solution for 5 min [30]. The limited amount of time with respect to the ammonia-based solution extraction was decided, in this case, after several tests on the indigo mock-up in order to prevent the formation of a salty halo on the archaeological sample, which is due to, probably, the presence of sodium dithionite.

### 4.5. Preparation of Ag-Colloidal Pastes

Ag-colloidal pastes were prepared by adapting the procedure already described in [52,53]. In brief, Ag-colloids were prepared following Leopold and Lendl’s methodology [54]: two solutions, one containing 0.021 g of NH_2_OH HCl in 5 mL of MilliQ water and the other one with 0.02 g of NaOH in 5 mL of MilliQ water, were added to a solution of 0.017 g of AgNO_3_ in 90 mL of MilliQ water under gentle magnetic stirring to induce the formation of colloids. Then, 10 mL of Ag-colloids were centrifuged for 20 min at 4500 rpm and the supernatant was removed. Afterwards, colloidal pastes were applied onto the gel surface immediately after dye extraction using a Pasteur pipette, and the gels were left drying for 12 h [30].

### 4.6. Spectroscopic Analysis

UV–Vis transmittance spectra were collected in the wavelength range between 190 and 800 nm using a Perkin Elmer Lambda 1050+ spectrophotometer in the ENEA C. R. Frascati laboratories. Measurements were led by housing the sample in a homemade support specifically designed for measurements on solid samples and by exposing both sides of the gel (i.e., the one in direct contact with the mockup where the extraction was performed and the opposite side) to the radiation. For each side, three measurements were acquired in three different positions. Spectra were then averaged and processed using Origin9 (©OriginLab, Northampton, MA, USA).

Raman-SERS data were collected using a Horiba Jobin-Yvon HR-Evolution Raman spectrometer (Kyoto, Japan) coupled with a microscope equipped with a series of interchangeable objectives. In this case, 20x magnification was chosen to select the area of analysis, while 100× objective was used to focus the laser beam on the Ag-colloid spots observed on the gels to obtain good quality SERS spectra. Samples were excited using a He–Ne laser (λ = 633 nm), whose intensity varied between 0.15 and 0.75 mW. The acquisition time and number of acquisitions were varied for each sample to optimize the signal-to-noise ratio; up to five spectra were acquired in different points of the gels and, to ensure reproducibility, data were averaged and processed using Origin9 (©OriginLab). Fifth-grade polynomial baseline was subtracted for the background and the adjacent–averaging smoothing method was applied to reduce noise.

Preliminary analysis was led on the archaeological fragment using a BELPhotonic optical microscope (Bengaluru, India) equipped with interchangeable objectives. After a general visual evaluation, fiber optic reflectance spectroscopy (FORS) measurements were performed to have a first non-invasive hypothesis of the chemical class to which natural dyes belong to better understand the procedure of extraction to follow. Spectra were acquired using the EXEMPLAR LS BW TECH spectrometer (Plainsboro Township, NJ, USA), operating in the range of 180–1100 nm with a variable resolution from 0.6 to 6.0 nm. Samples were illuminated with a 5W BW TECH BPS101 halogen lamp with an emission spectrum between 350 and 2600 nm and a color temperature of 2800 K. Radiation was sent to (and collected from) the samples using THORLABS RP22 optical fiber bundles provided with a measuring head of 45° inclination, which was suited to avoid the collection of specular reflectance radiation. Five measurements were acquired for each area (red and blue), and then spectra were averaged and processed using Origin9 (©OriginLab). The same instruments and methodology were used to evaluate chromatic variations in the mock-ups of yarns dyed with madder and the painting layer constituted by madder lake in egg tempera by exploiting the color analysis tool of the software BWSpec version 4.10. For the evaluation of the color invasiveness, further details about the experimental procedure are provided in [30].

## Data Availability

The data presented in this study are available on request from the corresponding author. The data are not publicly available due to the fact no actual database is available and they are part of a current project.

## Supplementary Materials

The following supporting information can be downloaded at: https://www.mdpi.com/article/10.3390/gels9070514/s1, Figure S1: The historical samples from Tutankhamun’s tomb analysed in the paper, constituted by archaeological textile fragments; Figure S2: A 10x magnification image of a red area on the analysed textile fragment; Figure S3: A 10x magnification image of a bluish area on the analysed textile fragment.
