# Supplementary material for: Applying Gel-Supported Liquid Extraction to Tutankhamun’s Textiles for the Identification of Ancient Colorants: A Case Study
Source: Gels. 2023 Jun 25;9(7):514. doi: 10.3390/gels9070514 (PMC10379361; doi:10.3390/gels9070514)
Supplement: Supplementary file 1 [file gels-09-00514-s001.zip › gels-2451305-supplementary.pdf]

Supplementary Materials

Applying Gel-Supported Liquid Extraction to Tutankhamun's Textiles for the Identification of Ancient Colorants: A Case Study

Greta Peruzzi [1,2,*], Alessandro Ciccola [3,*], Adele Bosi [3,4], Ilaria Serafini [3], Martina Negozio [5], Nagmeldeen Morshed Hamza [4,5,6], Claudia Moricca [5], Laura Sadori [5], Gabriele Favero [5], Valentina Nigro [7], Paolo Postorino [2] and Roberta Curini [3]

[1] Institute for Complex System, National Research Council, Sapienza University, Piazzale Aldo Moro 5, 00185 Rome, Italy
[2] Department of Physics, Sapienza University of Rome, Piazzale Aldo Moro 5, 00185 Rome, Italy; paolo.postorino@roma1.infn.it
[3] Department of Chemistry, Sapienza University of Rome, Piazzale Aldo Moro 5, 00185 Rome, Italy; adele.bosi@uniroma1.it (A.B.); ilaria.serafini@uniroma1.it (I.S.); roberta.curini@uniroma1.it (R.C.)
[4] Department of Earth Sciences, Sapienza University of Rome, Piazzale Aldo Moro 5, 00185 Rome, Italy; nagmeldeenmorshed.hamza@uniroma1.it
[5] Department of Environmental Biology, Sapienza University of Rome, Piazzale Aldo Moro 5, 00185 Rome, Italy; negozio.1920109@studenti.uniroma1.it (M.N.); claudia.moricca@uniroma1.it (C.M.); laura.sadori@uniroma1.it (L.S.); gabriele.favero@uniroma1.it (G.F.)
[6] Grand Egyptian Museum, Conservation Center, Al Remaya Square, Giza 3513204, Egypt
[7] ENEA C.R. Frascati, Fusion and Technologies for Nuclear Safety and Security Department, Via E. Fermi 45, 00044 Frascati, Italy; valentina.nigro@enea.it
* Correspondence: gretaperuzzi@icloud.com (G.P.); alessandro.ciccola@uniroma1.it (A.C.)

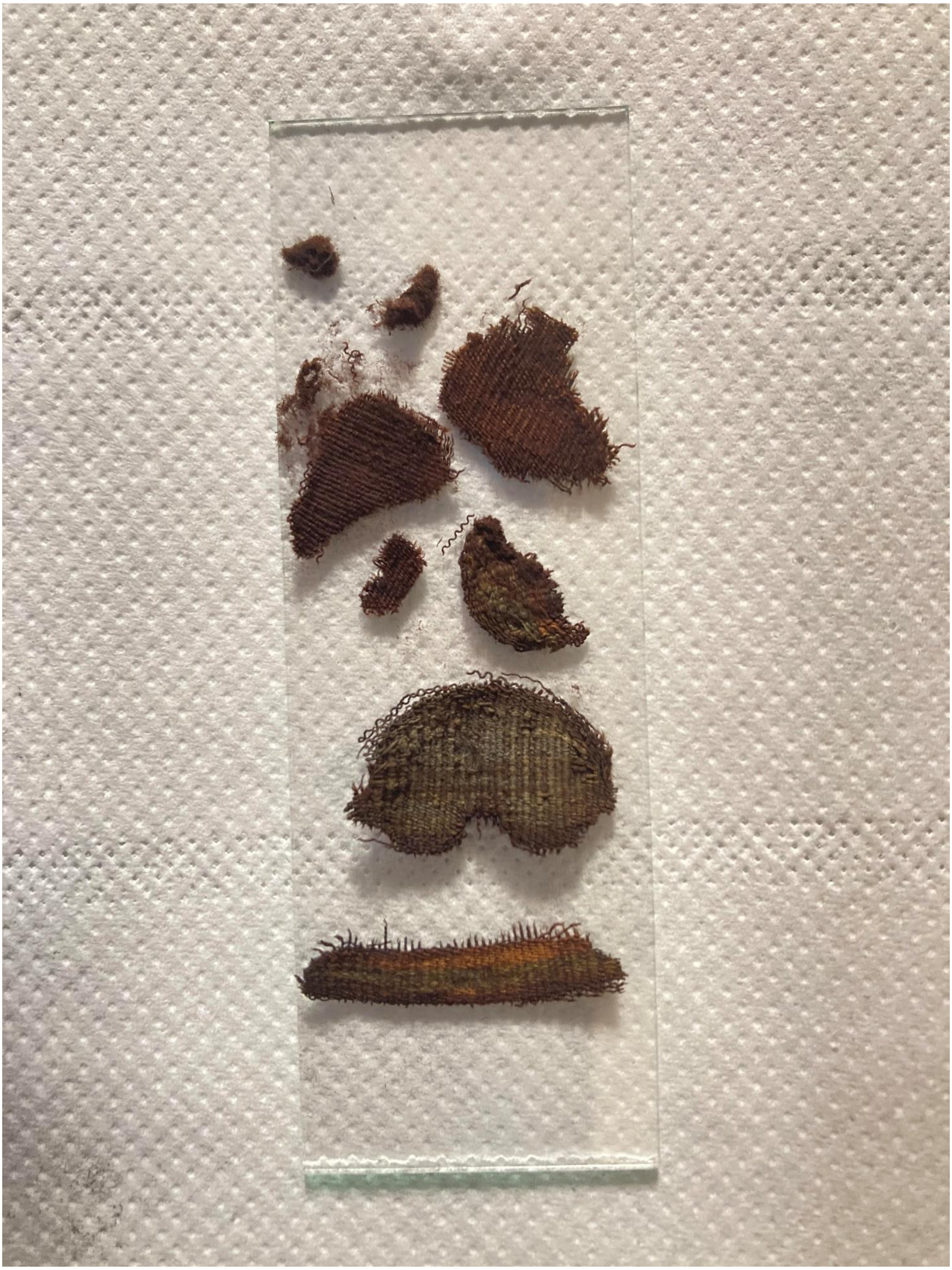

Figure S1: the historical samples from Tutankhamun's tomb analysed in the paper, constituted by archaeological textile fragments.

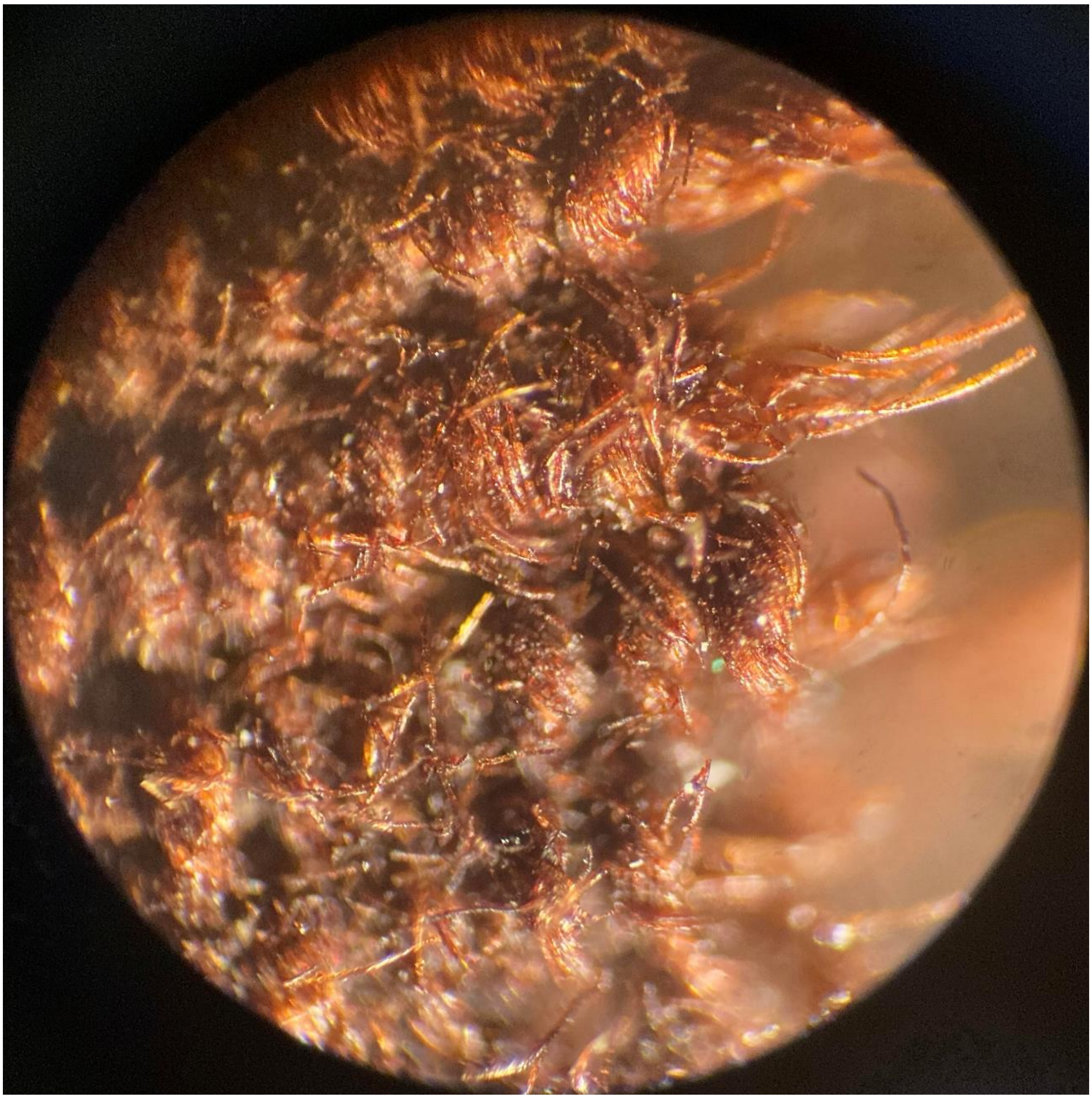

Figure S2: a 10x magnification image of a red area on the analysed textile fragment.

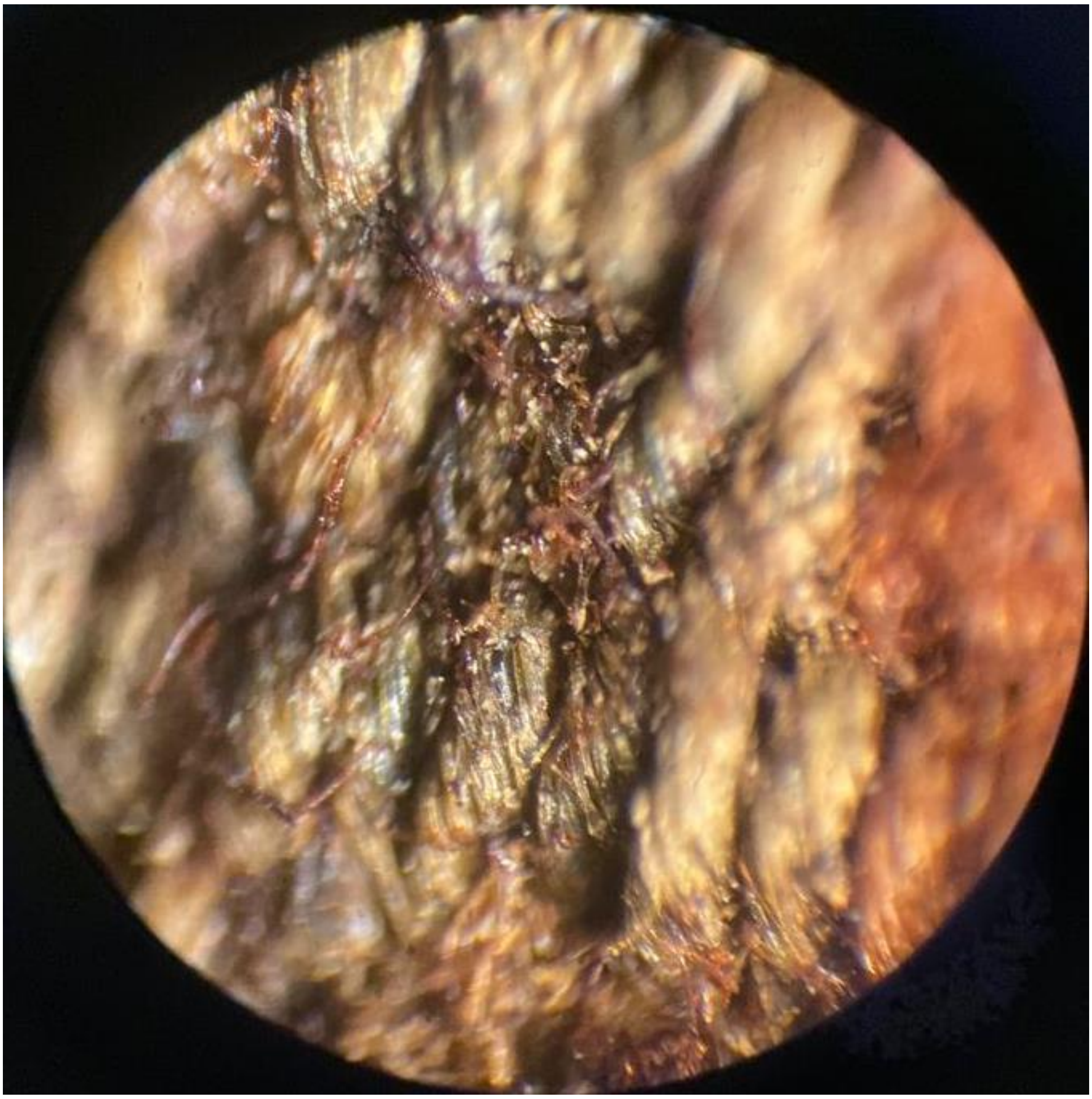

Figure S3: a 10x magnification image of a bluish area on the analysed textile fragment.
